# Self-reported symptoms among participants in a population-based screening program

**DOI:** 10.1016/j.breast.2020.08.015

**Published:** 2020-08-31

**Authors:** Marthe Larsen, Marie Lilleborge, Einar Vigeland, Solveig Hofvind

**Affiliations:** a Section for Breast Cancer Screening, Cancer Registry of Norway, Oslo, Norway; b Department of Radiology, Vestfold Hospital, Tønsberg, Norway; c Faculty of Health Sciences, Oslo Metropolitan University, Oslo, Norway

**Keywords:** Breast cancer, Screening, Symptoms, Interval breast cancer, Tumour characteristics, Lump, Breast neoplasm, Mass Screening, Signs and Symptoms, Prognosis

## Abstract

**Background:**

A limited number of studies have explored the association between self-reported symptoms and the risk of breast cancer among participants of population based screening programs.

**Methods:**

We performed descriptive statistics on recall, screen-detected and interval cancer, positive predictive value and histopathological tumour characteristics by symptom group (asymptomatic, lump, and skin or nipple changes) as reported from 785,642 women aged 50–69 when they attended BreastScreen Norway 1996–2016. Uni- and multivariable mixed effects logistic regression models were used to analyze the association between symptom group and screen-detected or interval cancer. Results were presented as odds ratios and 95% confidence intervals (CI).

**Results:**

A lump or skin/nipple change was reported in 6.2% of the 3,307,697 examinations. The rate of screen-detected cancers per 1000 examinations was 45.2 among women with a self-reported lump and 5.1 among asymptomatic women. Adjusted odds ratio of screen-detected cancer was 10.1 (95% CI: 9.3–11.1) and 2.0 (95% CI: 1.6–2.5) for interval cancer among women with a self-reported lump versus asymptomatic women. Tumour diameter, histologic grade and lymph node involvement of screen-detected and interval cancer were less prognostically favourable for women with a self-reported lump versus asymptomatic women.

**Conclusion:**

Despite targeting asymptomatic women, 6.2% of the screening examinations in BreastScreen Norway was performed among women who reported a lump or skin/nipple change when they attended screening. The odds ratio of screen-detected cancer was higher for women with versus without symptoms. Standardized follow-up guidelines might be beneficial for screening programs in order to take care of women reporting signs or symptoms of breast cancer when they attend screening.

## Highlights

• 6.2% of the exams were registered with symptoms in BreastScreen Norway.
• 45.2 per 1000 exams with a lump were diagnosed with screen-detected cancer.
• Tumor characteristics were less favourable for screen-detected cancer following a self-reported lump.
• 9.7 per 1000 exams with a lump, but no mammographic findings resulted in a screen-detected cancer.

## 1 Introduction

A lump in the breast is the most common symptom of breast cancer, and is often emphasized by breast cancer awareness campaigns [1]. Other breast cancer symptoms are skin and nipple changes, retraction, and nipple discharge. Breast cancer screening programs are aimed at asymptomatic, average-risk women, while women with lumps or other breast symptoms are recommended to seek medical advice [2]. However, some women report symptoms when they show up for screening in population-based programs [3,4]. A high proportion of symptomatic women might reduce the efficacy of a screening program, as women with symptomatic breast cancers are expected to have less prognostically favourable histopathological tumour characteristics compared to women with screen-detected cancer, and should have sought medical advice earlier [5,6]. Further, survival differ by detection mode among women with similar histopathologic tumour characteristics [7].

The number of studies on breast symptoms among screening participants and the risk of breast cancer, is limited. In Finland, at least one symptom (lump, scar, retraction, mole or nipple discharge) was reported at 25% of the screening examinations performed during the period from 2006 to 2010 [4]. A higher incidence of breast cancer and disease-specific mortality were reported among women with versus without symptoms [8,9]. Further, women with a lump or retraction had a higher risk of tumour diameter above 20 mm and histologically high-grade tumour compared to asymptomatic women [4].

Screening programs for breast cancer are moving towards stratification and individualized approaches. Several verified risk score models are available, and can be used to place women in screening schemes based on their estimated risk [[10], [11], [12]]. Lump, skin and nipple discharge, and other breast symptoms are known risk factors and signs of breast cancer. Knowledge about these factors among screening participants and the risk of breast cancer are less investigated, but might be important in the work towards risk stratification.

We wanted to take advantage of the data collected as a part of BreastScreen Norway, and explore self-reported symptoms and associated rates and risk of recall and breast cancer among women who have particicipated in the program. Further, we wanted to analyze histopathologic characteristics of the tumours among women who did and did not report a symptom and were diagnosed with breast cancer.

## 2 Materials and methods

In this register-based study, we used data from BreastScreen Norway, from the period 1996–2016. BreastScreen Norway is a population-based screening program, offering women aged 50–69 two-view mammography biennially. The screening examinations took place at 26 stationary and 4 mobile units, while screen-reading, further assessment and eventual treatment of women with breast cancer were performed at 16 breast centers. Annual attendance rate was about 76%, and 84% of the invited women had attend at least once during the study period [13]. As a part of their screening examination, the women underwent a short interview by a radiographer before the imaging. At the interview, the women were asked about symptoms, or whether they had any changes in the breast during tha last months. The radiographers registered the women’s response in a predefined form, according to local procedures. Standard procedure for screen-reading was independent double reading by breast radiologists. Each breast was assigned a score of 1–5 by each radiologist (1, negative for malignancy; 2, probably benign; 3, intermediate suspicion of malignancy; 4, probably malignant; 5, high suspicion of malignancy). If either radiologist assigns a score of 2 or higher, a consensus meeting determines whether to recall the woman for further assessment. If the two radiologist do not agree, final decision on recall is made in a consensus meeting or a third radiologist act as an arbitrator. The Cancer Registry of Norway administers the program, while the Cancer Registry Regulation ensure approval with waiver of informed consent to perform surveillance, quality assurance, and studies on the basis of data collected as a part of the program [14]. The program is described in detail elsewhere [13].

**Fig. 1:**
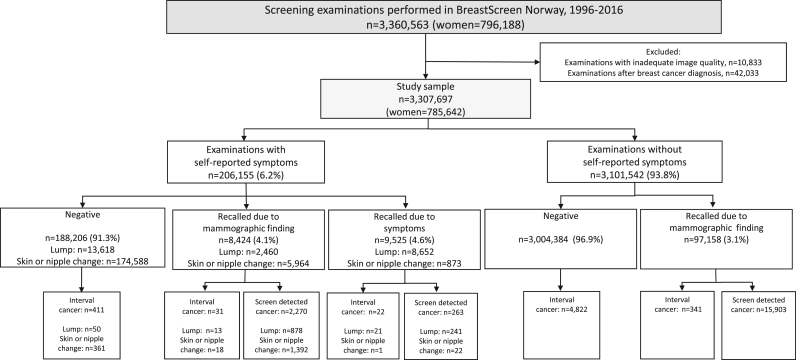
Flow chart, final study sample and screening outcome by symptoms versus no symptoms.

We obtained a pseudonymized data file with information on 3,360,563 screening examinations performed among 796,188 women during the study period (Fig. 1). Examinations with inadequate image quality (n = 10,833) and examinations on women with a history of breast cancer (n = 42,033) were excluded. As a result, the final study sample included 3,307,697 screening examinations from 785,642 women.

### 2.1 Variables of interest

Information about breast symptoms reported by the women at the pre-screening interview were registered in the form or as comments, which were available for the screen-readers. We classified the information from the form into three groups; asymptomatic (no symptoms), lump (palpable lump or tumour), and skin/nipple changes (nipple discharge, retraction, peau d’orange or skin changes). Moles, scars, warts and eczema were reported, but included in the asymptomatic group in this analysis. If both lumps and skin/nipple changes were registered at one examination, we classified it as a lump. All examinations registered with a lump were flagged for the radiologists at screen-reading and were automatically selected for discussion at the consensus meeting. However, this information could be overruled by the radiologists and the screening examination could be interpreted as negative, or as a mammographic finding (score 2 or higher).

Our data file also included information about age at screening and screening outcome. Screening outcome included recalls, screen-detected and interval cancer, histologic type, tumour diameter, histologic grade, lymph node involvement, and estrogen and progesterone receptor status. A recall could be due to mammographic findings (mammographic recall), or due to symptoms (symptomatic recall). Examinations with self-reported symptoms, where the radiologists also identified mammographic abnormalities, were classified as mammographic recalls. Both ductal carcinoma in situ and invasive breast cancer were defined as breast cancer. A screen-detected cancer was defined as breast cancer diagnosed as a result of a recall and further assessment. An interval cancer was defined as breast cancer either diagnosed up to 24 months after a negative screening or 6–24 months after a false-positive screening result. For women diagnosed with interval cancer, we used reported symptom information given at the screening examination prior to the diagnosis.

### 2.2 Statistical analysis

Descriptive statistics was presented for recall, screen-detected, and interval breast cancer, and positive predictive value of recalls (PPV), stratified by symptom group. We defined symptom group for each examination, which means the individual woman could change symptom group for each examination. The PPV was calculated as the proportion of screen-detected cancers among recalls. Frequencies and proportions or median and interquartile range (IQR) were presented for all histopathologic tumour characteristics of invasive screen-detected and interval cancer, stratified by symptom group.

The association between breast cancer and self-reported symptoms was analyzed using uni- and multivariable mixed effects logistic regression with symptom group as the exposure. The assumption of independent observations in standard logistic regression was violated due to the repeated measures for each woman, and a random effect was included for the unique woman identifier. Results were presented as odds ratio (OR) with 95% confidence intervals (CI). Due to possible confounding, the ORs were adjusted for age and screening history (Categorical variable: First/not first screening examination) in the multivariable analysis.

In the supplemental material, we present descriptive statistics by breast center. Further, to widen our approach, we used a “never-ever approach” regarding reported symptoms and considered the women asymptomatic until her first reported symptom, which means that all examinations after reporting a symptom were included in the symptom group.

Stata 16 MP (StataCorp. 2019. Stata Statistical Software: Release 16. College Station, TX: StataCorp LLC) was used to analyze the data.

## 3 Results

Among 3,307,697 screening examinations, a symptom was reported at 206,155 (6.2%) (Fig. 1). A lump was reported at 24,730 (0.7%) and skin or nipple changes at 181,425 (5.5%) of the examinations (Table 1). We found substantial variation in the distribution of symptom groups between the breast centers in BreastScreen Norway (Tables S–1).

**Table 1:** Frequencies (n) and rates of recalls (%), screen-detected and interval cancer per 1000 screening examinations, and positive predictive value (PPV) stratified by symptom group.

Indicators	Symptom group			
	Total 3,307,697 (100%)	Lump n = 24,730 (0.7%)	Skin or nipple changes n = 181,425 (5.5%)	Asymptomatic n = 3,101,542 (93.8%)
Recalls, n (%)				
Total	115,107 (3.5)	11,112 (44.9)	6837 (3.8)	97,158 (3.1)
Mammographic findings	105,582 (3.2)	2460 (9.9)	5964 (3.3)	97,158 (3.1)
Symptoms	9525 (0.3)	8652 (35.0)	873 (0.5)	–
Screen-detected cancer, n (rate per 1000 screening examinations)				
Total	18,436 (5.6)	1119 (45.2)	1414 (7.8)	15,903 (5.1)
Mammographic findings	18,173 (5.5)	878 (35.5)	1392 (7.7)	15,903 (5.1)
Symptoms	263 (0.1)	241 (9.7)	22 (0.1)	–
Interval cancer, n (rate per 1000 screening examinations)				
Total	5627 (1.7)	84 (3.4)	380 (2.1)	5163 (1.7)
Mammographic findings	372 (0.1)	13 (0.5)	18 (0.1)	341 (0.1)
Symptoms	22 (−)	21 (0.8)	1 (−)	–
Negative	5233 (1.6)	50 (2.0)	361 (2.0)	4822 (1.6)
PPV, screen-detected cancer/recall (%)				
Total	18,436/115,107 (16.0)	1119/11,112 (10.1)	1414/6837 (20.7)	15,903/97,158 (16.4)
Mammographic findings	18,173/105,582 (17.2)	878/2460 (35.7)	1392/5964 (23.3)	15,903/97,158 (16.4)
Symptoms	263/9525 (2.8)	241/8652 (2.8)	22/873 (2.5)	–

The overall recall rate during the study period was 3.5%: 3.2% due to mammographic recall and 0.3% due to symptomatic recall (Table 1). Among screening examinations where a lump was reported, 44.9% were recalled: 9.9% due to mammographic findings and 35.0% due to symptoms. The rest was deselected at the consensus meeting. Among screening examinations where skin/nipple changes were reported, 3.8% were recalled: 3.3% due to mammographic findings and 0.5% due to symptoms. Among the asymptomatic, 3.1% were recalled.

The overall rate of screen-detected cancers was 5.6 per 1000 screening examinations (Table 1). For examinations registered with a symptom, the rate was 12.3 per 1000 while it was 5.1 per 1000 for asymptomatic examinations. The rate of screen-detected cancers where a lump was reported was 45.2 per 1000 examinations; 35.5 per 1000 among those with mammographic findings and 9.7 per 1000 for examinations where the woman reported a lump and with no mammographic findings. Further, the rate was 7.8 per 1000 for skin/nipple changes and 5.7 per 1000 for asymptomatic examinations. For examinations where a lump was reported, the PPV was 35.7% for mammographic recalls and 2.8% for symptomatic recalls.

The overall interval cancer rate was 1.7 per 1000 screening examinations (Table 1). For examinations where a lump was reported, the rate was 3.4 per 1000, while it was 2.1 per 1000 for skin/nipple changes, and 1.7 per 1000 for asymptomatic screening examinations (Table 1).

The median tumour diameter for invasive screen-detected cancers was 19 mm (IQR: 13–29) among those who reported a lump, 15 mm (IQR: 10–21) for skin/nipple changes and 12 mm (IQR: 9–18) for screen-detected cancers among asymptomatic women (Table 2a). We identified lymph node involvement in 41.0% of the screen-detected cancer following an examination with a lump, and in 28.4% and 20.6% of the cases detected after an examination where skin/nipple changes were reported and no symptoms reported, respectively.

**Table 2a:** Histopathologic tumor characteristics of screen-detected cancer stratified by symptom group. Variables are presented with frequencies (n) and percentage (%) or median with interquartile range (IQR).

	Symptom group		
	Lump	Skin or nipple changes	Asymptomatic
	n = 1119	n = 1414	n = 15,903
Histologic type, n (%)			
Ductal carcinoma in situ	74 (6.6)	206 (14.6)	3011 (18.9)
Invasive breast cancer	1045 (93.4)	1208 (85.4)	12,892 (81.1)
Invasive breast cancer (n = 15,145)			
Tumor diameter, mm, median (IQR)	19 (13–29)	15 (10–21)	12 (9–18)
Information not available, n	74	31	247
Histologic grade, n (%)			
1	221 (21.8)	350 (29.6)	4243 (33.6)
2	487 (48.0)	594 (50.3)	6087 (48.2)
3	306 (30.2)	237 (20.1)	2312 (18.3)
Information not available, n	31	27	250
Positive lymph nodes, n (%)	419 (41.0)	328 (28.4)	2603 (20.6)
Information not available, n	24	17	242
Positive estrogen receptor status, n (%)	852 (84.8)	1052 (90.9)	11,090 (89.6)
Information not available, n	40	50	513
Positive progesterone status, n (%)	658 (65.7)	846 (73.7)	8793 (71.6)
Information not available, n	43	60	614

The median tumour diameter for interval cancers was 19 mm (IQR: 13–30) when a lump was reported, 19 mm (IQR: 12–25) for skin/nipple changes and 18 mm (IQR: 12–25) for those detected among asymptomatic women (Table 2b). Lymph node involvement was observed in 31.9% of the interval cancers after an examination where a lump was reported, 40.1% when skin/nipple changes were reported and 39.6% where no symptoms were reported.

**Table 2b:** Histopathologic tumor characteristics of interval cancer stratified by symptom group. Variables are presented with frequencies (n) and percentage (%) or median with interquartile range (IQR).

	Symptom group		
	Lump	Skin or nipple changes	Asymptomatic
	n = 84	n = 359	n = 4878
Histologic type, n (%)			
Ductal carcinoma in situ	10 (11.9)	21 (5.5)	285 (5.5)
Invasive breast cancer	74 (88.1)	359 (94.5)	4878 (94.5)
Invasive interval cancer (n = 5311)			
Tumor diameter, mm, median (IQR)	19 (13–30)	19 (12–25)	18 (12–25)
Information not available, n	5	34	469
Histologic grade, n (%)			
1	15 (20.8)	55 (15.9)	783 (16.7)
2	36 (50.0)	172 (50.0)	2164 (46.0)
3	21 (29.2)	118 (34.2)	1757 (37.4)
Information not available, n	2	14	174
Lymph node involvement, n (%)	23 (31.9)	137 (40.1)	1864 (39.6)
Information not available, n	2	17	173
Positive estrogen receptor status, n (%)	58 (81.7)	274 (79.4)	3631 (77.2)
Information not available, n	3	14	175
Positive progesterone status, n (%)	51 (71.8)	214 (62.4)	2704 (58.0)
Information not available, n	3	16	212

Adjusted OR for screen-detected cancer among women who reported a lump was 10.1 (95% CI: 9.3–11.1) and 1.5 (95%CI: 1.4–1.6) for those who reported skin/nipple (Table 3). For interval cancers, the adjusted OR was 2.0 (95%CI: 1.6–2.5) when a lump was reported.

**Table 3:** Unadjusted and adjusted odds ratio (OR) for screen-detected and interval breast cancer with 95% confidence interval (CI). Fixed effect adjustment variables were age and prevalent/incident screening examination.

	Unadjusted OR (95% CI) of screen-detected cancer	Adjusted OR (95% CI) of screen-detected cancer	Unadjusted OR (95% CI) of interval cancer	Adjusted OR (95% CI) of interval cancer
Asymptomatic	Reference	Reference	Reference	Reference
Skin or nipple changes	1.5 (1.4–1.6)	1.5 (1.4–1.6)	1.3 (1.1–1.4)	1.3 (1.1–1.4)
Lump	9.2 (8.6–9.8)	10.1 (9.3–11.1)	2.0 (1.6–2.5)	2.0 (1.6–2.5)

Using the never-ever approach, OR for screen-detected cancer following an examination where a lump was reported was 5.1 (95% CI: 4.7–5.3) (Tables S–2). The distribution of histopathologic tumour characteristics of screen-detected and interval cancers using the same approach is shown in Table S-3a and S-3b.

## 4 Discussion

In this registry-based study using data from BreastScreen Norway, the odds of screen-detected cancer among women reporting a lump when they attended screening was 10 times higher than for asymptomatic women. For every 1000 screening examinations where a lump was reported we identified 45.2 screen-detected cancers and 3.4 interval cancers. The rates of screen-detected and interval cancer were 5.1 and 1.7, respectively, per 1000 examinations among asymptomatic women. The rate of screen-detected cancer among women reporting a lump, but without mammographic findings, was 9.7 per 1000 examinations. However, the majority of the screen-detected cancers among symptomatic women were diagnosed after a recall due to mammographic findings. Histopathological tumour characteristics were less prognosticcally favourable for women with screen-detected cancers after self-reported symptoms, compared to screen-detected cancers among asymptomatic women.

Breast cancer screening programs are designed for asymptomatic women with average risk of breast cancer [2]. The substantially higher risk of screen-detected and interval cancer among women reporting a lump when they show up for screening indicate that women with an increased risk of breast cancer participate in the program. This finding is supported by the histopathologic tumour characteristics of the screen-detected cancers found among these women, which were comparable to characteristics of tumours detected outside of the screening program [4,6,15]. We assume that women reporting a lump when they attend screening have been aware of the lump for a while but waited for the invitation, which they knew would come within a two-year period, to disclose the lump to the health services. BreastScreen Norway recommends women to seek their general practitioner if they have breast symptoms. These recommendations are available in the information leaflet all women receive together with the invitation to the screening program, on the program’s website, and are also given orally to the attending women by the radiographers working at the screening unit. However, our findings underscore the significance of continuously communicating the importance of an appointment with a doctor if a lump or other changes in the breasts appear.

A study from the UK found that information about symptoms had little bearing on the decision of recall for further assessment and that breast cancer was uncommon among women with symptoms in combination with negative mammograms [16]. The recall rate for women with a lump and no mammographic findings was 35% in our study, which is in contrast to the findings in the UK. However, we found a substantial variation across breast centers regarding procedures on how and to which degree information about symptoms was used in the screen-reading. Due to the flagging, we presume the radiologists’ awareness was drawn to these examinations. The high PPV for mammographic recall among examinations where a lump was reported might be due to the combination of the flagged symptoms and the radiologists’ interpretation of the mammograms.

With current procedures for registration of self-reported symptoms in BreastScreen Norway and based on our findings, a policy of recalling women based solely on a self-reported symptom is not justifiable. However, ethical aspects have to be included in the evaluation of the numbers. Despite a low PPV for women reporting lumps and symptomatic recall, it is challenging to argue against the recalling of the women. These women might expect to be recalled for further assessment to rule out malignancy in the lump. In this way, a “false positive” screening examination might be reassuring for these women.

Women reported a lump in 0.7% of the screening examinations in our study. The proportion was two times lower than what was found in the Finnish screening program [4,8,9]. However, the rate of screen-detected cancers among women who reported a lump was lower in Finland than in Norway. We included lumps only if it was registered on the standardized form, and not if it was reported as a comment, which might underestimate the number and explain the difference between the two countries. On the other hand, we observed a substantial variation in the distribution of lumps and skin/nipple changes between the breast centers. Standardized forms and procedures for the pre-screening interviews including regular training for the radiographers, who register the information, might reduce this variation, and thus improve the quality of the screening program.

The trend towards stratified and individualized screening for breast cancer aims to increase the benefit and reduce the harms for the women and for the society [10,11]. Breast cancer diagnosed after a lump was shown to be diagnosed at a more advanced stage and to be less prognostically unfavourable compared with asymptomatic screen-detected breast cancer. A current randomized controlled trial is designed to compare risk-stratified screening and standard mammographic screening [12]. Prior breast biopsies are included in the risk assessment used for stratification, but not symptoms such as lumps or skin/nipple changes alone. When stratifying by giving the women individual screening intervals it might be even more significant to communicate the importance of visiting a doctor if symptoms appear between screening intervals. Another aspect is including symptom information in the risk model and stratification. However, discriminating between the high risk and the low risk symptoms represent a challenge.

Women reporting a lump had an increased risk of interval cancer compared to asymptomatic women. However, the percentage of invasive interval cancer following a lump was lower than for skin/nipple changes and for asymptomatic women, and we found histopathologic favourable tumour characteristics among those detected after a lump compared to those detected after skin/nipple changes and no symptoms. However, the number of interval cancers following a lump was small and we do not know if the tumours associated with interval cancers were missed, misinterpreted or were in the same breast as the reported lump. These factors represent limitations of the study.

Further limitations were related to the registration of the symptoms. In addition to a pre-defined form, the radiographers had the possibility to give comments in a text-field. This information was not included in this study. Further, attendance bias might represent a challenge since an invitation might cause breast awareness and a self-examination, resulting in the woman finding a lump or skin/nipple change. These women are probably more likely to accept the invitation compared to those without any symptoms. Due to inconsistent reporting and variation within symptom groups, our findings must be interpreted carefully. The strengths of our study were the large study sample from a population based screening program over two decades. Further, the registration system has not changed during the study period.

In conclusion, despite the fact that BreastScreen Norway targets asymptomatic women, 6.2% showed up with symptoms at their screening examination. The odds of screen-detected cancer was 10 times higher for screening examinations where a lump was reported compared to examinations where no symptoms were reported. Standardized follow-up guidelines might be beneficial for screening programs in order to take care of women reporting signs or symptoms of breast cancer when they show up for screening.
