# Supplementary material for: Self-reported symptoms among participants in a population-based screening program
Source: Breast. 2020 Aug 31;54:56–61. doi: 10.1016/j.breast.2020.08.015 (PMC7495098; doi:10.1016/j.breast.2020.08.015)
Supplement: Multimedia component 1 [file mmc1.docx]

Table S-1: Frequency (n) and percentage (%) of reported symptoms in
each symptom group by breast center in BreastScreen Norway, 1996-2016*.
Highest and lowest percentage are in bold

| **Breast Center # Symptom group** | | | | |
| --- | --- | --- | --- | --- |
|  | Lump | Skin or nipple changes | Asymptomatic | Total |
| 1 | **1,555 (0.3)** | 23,013 (6.9) | 311,040 (92.8) | 335,208 |
| 2 | 2,843 (0.8) | 16,288 (4.4) | 354,664 (94.9) | 373,795 |
| 3 | 2,093 (0.8) | 10,085 (2.9) | 334,312 (96.3) | 347,300 |
| 4 | 1,985 (1.0) | 11,937 (5.9) | 189,162 (93.1) | 203,084 |
| 5 | 46 (0.04) | **1,849 (1.4)** | 128,130 (98.5) | 130,025 |
| 6 | **564 (0.3)** | 9,282 (4,8) | 184,517 (94.9) | 194,363 |
| 7 | 959 (0.6) | 10,427 (6,3) | 155,255 (93.2) | 166,641 |
| 8 | 658 (0.4) | 5,100 (2.9) | 170,133 (96.7) | 175,891 |
| 9 | 798 (0.5) | 12,981 (8.1) | 146,381 (91.4) | 160,160 |
| 10 | 1,479 (1.3) | 5,819 (5.3) | **102,719 (96.7)** | 110,017 |
| 11 | **4,833 (1.8)** | 17,408 (6.4) | 249,467 (91.8) | 271,708 |
| 12 | 1,155 (1.0) | 10,186 (8.4) | **110,057 (90.7)** | 121,398 |
| 13 | 882 (0.6) | 6,710 (4.9) | 129,731 (94.5) | 137,323 |
| 14 | 579 (0.9) | 3,541 (5.1) | 63,435 (94.0) | 67,465 |
| 15 | 680 (0.5) | 6,712 (5.2) | 122,145 (94.3) | 129,537 |
| 16 | 715 (0.6) | **9,471 (8.5)** | 101,162 (90.9) | 111,348 |
| 17 | 926 (0.7) | 10,574 (7.5) | 130,115 (91.9) | 141,615 |
| 18 | 263 (1.0) | 1,961 (7.4) | 24,381 (91.6) | 26,605 |
| 19 | 1,307 (1.3) | 8,171 (7.8) | 94,736 (90.9) | 104,214 |

* The number of breast centers of today is 17, since some are merged during the study period

Table S-2: Total number of screening examinations, screen-detected cancer (SDC) and interval cancer (IC), and unadjusted/adjusted odds ratio (OR) of SDC and IC with 95% confidence interval (CI), by symptom group

| Symptom group | Examin-  ations | SDC | IC | Unadjusted OR (95% CI) of SDC | Adjusted OR (95% CI) of SDC | Unadjusted OR (95% CI) of IC | Adjusted OR (95% CI) of IC |
| --- | --- | --- | --- | --- | --- | --- | --- |
| Never reported a symptom | 3,055,508 | 15,653 | 4,622 | Reference | Reference | Reference | Reference |
| Ever reported skin or nipple changes | 176,442 | 1,381 | 487 | 1.6 (1.5-1.7) | 1.6 (1.5-1.7) | 1.3 (1.1-1.4) | 1.3 (1.1-1.4) |
| Ever reported a lump | 75,747 | 1,402 | 250 | 4.7 (4.3-5.0) | 5.1 (4.7-5.3) | 2.0 (1.8-2.3) | 2.0 (1.8-2.3) |

Table S-3a: Histopathologic tumor characteristics of invasive screen-detected breast
cancer (SDC) stratified by symptom group. Variables are presented with frequencies (n)
and percentage (%) or median and interquartile range (IQR)

|  | **Symptom group** | | |
| --- | --- | --- | --- |
|  | Ever reported a lump | Ever reported skin or nipple change | Never  reported any symptoms |
|  | n=1,402 | n=1,675 | n=15,359 |
| Histologic type, n (%) |  |  |  |
| Ductal carcinoma *in situ* | 117 (8.4) | 250 (14.9) | 2,924 (19.0) |
| Invasive | 1,285 (91.7) | 1,425 (85.1) | 12,435 (81.0) |
| **Invasive SDC (n=15,145)** |  |  |  |
| Tumor diameter in mm, median (IQR) | 18 (12-25) | 14 (10-20) | 12 (9-18) |
| Information not available, n | 76 | 36 | 240 |
| Histologic grade, n (%) |  |  |  |
| 1 | 289 (23.1) | 428 (30.7) | 4,097 (33.6) |
| 2 | 602 (48.1) | 695 (49.9) | 5,871 (48.2) |
| 3 | 360 (28.8) | 271 (19.4) | 2,224 (18.2) |
| No information | 31 | 34 | 243 |
| Positive lymph nodes, n (%) | 460 (36.6) | 376 (26.8) | 2,524 (20.7) |
| Information not available, n | 27 | 21 | 235 |
| Positive estrogen receptor status, n (%) | 1,058 (85.5) | 1,238 (90.4) | 10,698 (89.6) |
| Information not available, n | 48 | 55 | 500 |
| Positive progesterone status, n (%) | 823 (66.8) | 1,001 (73.7) | 8,473 (71.6) |
| Information not available, n | 53 | 67 | 597 |

Table S-3b: Histopathologic tumor characteristics of invasive interval breast cancer (IC)
stratified by symptom group. Variables are presented with frequencies (n) and percentage (%)
or median and interquartile range (IQR)

|  | **Symptom group** | | | |
| --- | --- | --- | --- | --- |
|  | Ever reported a lump | Ever reported skin or nipple change | Never  reported any symptoms |  |
|  | n=250 | n=487 | n=4,622 |  |
| Histologic type, n (%) |  |  |  |  |
| Ductal carcinoma *in situ* | 21 (8.4) | 27 (5.5) | 268 (5.5) |  |
| Invasive | 229 (91.6) | 460 (94.5) | 4,622 (94.5) |  |
| **Invasive IC (n=5,311)** |  |  |  |  |
| Tumor diameter in mm, median (IQR) | 19 (13-28) | 19 (12-25) | 18 (12-25) |  |
| Information not available, n | 17 | 48 | 443 |  |
| Histologic grade, n (%) |  |  |  |  |
| 1 | 39 (17.7) | 73 (16.5) | 741 (16.6) |  |
| 2 | 108 (49.1) | 223 (50.3) | 2,041 (45.8) |  |
| 3 | 73 (33.2) | 147 (33.2) | 1,676 (37.6) |  |
| Information not available, n | 9 | 17 | 164 |  |
| Lymph node involvement, n (%) | 75 (33.9) | 172 (39.1) | 1,777 (39.9) |  |
| Information not available, n | 8 | 20 | 164 |  |
| Positive estrogen receptor status, n (%) | 186 (83.4) | 355 (80.0) | 3,422 (76.9) |  |
| Information not available, n | 7 | 16 | 169 |  |
| Positive progesterone status, n (%) | 150 (68.5) | 276 (62.4) | 2,543 (57.6) |  |
| Information not available, n | 10 | 18 | 203 |  |
